# The Late Dr. E. S. Gaillard

**Published:** 1885-03

**Authors:** 


### THE LATE DR. E. S. GAILLARD.

Our exchanges announce the demise of Dr. E. S. Gaillard, who died at his home near New York, February 1, 1885.

Dr. Gaillard descended from highly honored Huguenot ancestry; was born in Charleston, S. C., in 1827. His literary and professional education was obtained in that State. Sharing fully the political opinions and patriotic impulses of the people of the South, in 1861 he entered the army of the Confederate States with the rank of surgeon. Many Southern physicians remember with pleasure their acquaintance with him, beginning on the plains of Manassas, and extending through the stirring scenes which preceded the battle of Seven Pines, in front of Richmond. In that engagement Dr. Gaillard received a wound which rendered necessary the amputation of his right arm. This, of course, ended his active duties in the field, but did not terminate his services to the Confederacy in other capacities; especially as inspector of hospitals, he was both active and efficient, and gained the respect and esteem of large numbers of the medical staff of the Confederate armies, who bear willing testimony to his capacity, fidelity to public trusts, personal honor and gentlemanly demeanor.

Since the close of the civil war Dr. Gaillard's efforts have been mainly in the field of medical literature. These have been too recent and their results too well known to require extended notice. The publication of the journal which bears his name has monopolized much of his time and thoughts. Since he began its publication in New York a few years ago, it has attained a circulation popularity and influence almost phenomenal. The old friends and confreres of the editor were extremely gratified at the success of "Gaillard's Medical Journal," and indulged the hope that it would become a permanent source of honorable competency for his declining years.

### ITEMS.

DR. W. P. HARDEN, of Smyrna, has removed to Harmony Grove.

MOST of the American Medical Colleges have had smaller classes than usual during the winter.

DR. HENRY WILE, a prominent physician of Philadelphia, is in the city visiting his friend, Dr. W. S. Elkin.

DR. J. H. LOGAN, of this city, has been very ill with pneumonia. We are glad to state that he is convalescent.

MRS. CLARISSA C. PECK, who recently died in Chicago, left $600,000 to found a home for incurables in that city.

DR. D. W. HAMMOND, of Macon, says that a saturated solution of sulphate of magnesia in water, is an excellent local application in erysipelas.

Dr. A. W. CALHOUN, of this city, spent several days in Augusta recently in attendance upon a meeting of the Directors of the Georgia Railroad.

DR. R. C. EVE, of Augusta, Ga., Professor Materia Medica in the Medical Department of the University of Georgia, died at his home January 30th.

THE annual meeting of the Medical Association of Georgia will be held in Savannah on the 15th, 16th and 17th of April next. Every physician in the State ought to be a member of this body.

WE have received from the publishers, Messrs. Ludden & Bates, Savannah, their latest publication, "Reunion Medley," an instrumental composition arranged by J. A. Bates for piano or parlor organ. It is inscribed "To Grover Cleveland, the people's choice." Mailed, postpaid, by the publishers for 25 cents.

DR. HOWARD J. WILLIAMS, of Macon, will deliver the annual oration at the approaching meeting of the Medical Association of Georgia, in Savannah.

THE MORNING NEWS, of Savannah, comes regularly to our table. It is one of the most reliable papers published in the State, and is ably edited.

DR. CARTER, of New York, a graduate of the University of New York, is visiting friends in this city. The Doctor visits Atlanta with a view of locating here.

THE Texas *Courier-Record of Medicine*, one of the brightest and best of our exchanges, has an advertisement in this issue, to which the attention of our readers is invited.

Dr. William Braithwaite, of Leeds, England, the founder and senior editor of the "*Retrospect of Medicine*," died January 31st, in the seventy-eighth year of his age.

WE take pleasure in announcing that *Gaillard's Medical Journal* will be continued with M. E. and E. W. Gaillard as publishers, with an able corps of collaborators.

DR. JOHN F. LANCASTER, of this city, has removed to Walden, Ga. Dr. Lancaster graduated from the Atlanta Medical College two years ago with honors, and has since that time been practicing in this city.

OUR readers would confer a very great favor on the publishers of THE JOURNAL if they would always mention THE JOURNAL in writing advertisers. This will cost them nothing, and will be worth a great deal to the publishers.

WE have received the twenty-third report of the Board of Trustees and Officers of the "Georgia Institution for the Education of the Deaf and Dumb" at Cave Springs. The report of the physician, Dr. Wright, shows the sanitary condition of the institution to be excellent.

THE thirty-sixth annual session of the American Medical Association will be held in New Orleans, La., on Tuesday, Wednesday, Thursday and Friday, April 28, 29, 30, and May 1, commencing on Tuesday at 11 a. m.

WE are in receipt of the eleventh annual report of the Superintendent of the Cincinnati Sanitarium for the year ending November 30th, 1884. One hundred and thirty-five new patients were admitted during the year.

SOME months ago Dr. Robert Battey, of Rome, while dressing a wound inoculated himself accidentally and has suffered considerably from it. We had a pleasant call from him a few days ago, and are pleased to state that he has entirely recovered.

DR. CHRISTOPHER C. GRAHAM, of Louisville, Ky., died on the evening of February 3d, after a short illness. He was one hundred years old last October. He was born in an old fort near Danville, Ky., and was an associate of Daniel Boone.

REPORTS of surgical operations upon the gall-bladder or ducts, whether previously published or not, are desired for a contribution to an important work; and all facts connected with them will be thankfully received by Dr. J. McF. Gaston, Atlanta, Ga.

DR. R. O. COTTER has located in Macon. Dr. Cotter was associated for four years with Dr. Calhoun, of this city, in the practice of diseases of the eye, ear and throat, and was also assistant to the chair of eye, ear and throat diseases in the Atlanta Medical College.

DR. HOWARD J. WILLIAMS, of Macon, was married January 29th, to Miss Kittie Jewett, daughter of Mr. H. L. Jewett, of Macon. Dr. Williams is one of the most prominent young physicians in the State. He is Secretary of the Macon Medical Society, and a member of the State Medical Association. We extend to the happy pair our congratulations.

A MICHIGAN physician pulls teeth for the mumps. He doubtless believes that the microbe of mumps adheres to the roots of the teeth.—*Savannah News.*

WE had a pleasant call a few weeks since from Mr. W. R. Wells, of Macon, representing Goodwyn & Small, proprietors and manufacturers of Goodwyn's Compound Syrup of Hypophosphites. Mr. Wells is now in the West, and we are glad to know is having good sales for this excellent preparation.

ONE HUNDRED YEARS OF PUBLISHING is the title of a handsomely printed little book which we have received from the well known medical publishers, Messrs. Lea Brothers & Co., of Philadelphia, giving a history of their house for the past hundred years. Like all works sent out by this house, it is done in the highest style of the art.

THE *Annals of Surgery*, a monthly review of surgical science and practice, edited by L. S. Pilcher, M. D., of Brooklyn, N. Y., and C. B. Keetley, F. R. C. S., of London, England, has made its appearance. It is the only journal in the English language devoted exclusively to surgery; is published simultaneously in the United States and Great Britain, and aims to become an invaluable part of the library of every working surgeon. The first and second numbers have been received. The journal presents a handsome appearance and is ably edited.

DR. R. F. WRIGHT, of Dalton, died in this city February 23d.

Dr. Wright graduated from the Medical Department of the University of Louisiana and spent several years in the hospitals of New Orleans. He afterwards spent two years in Europe. Returning from Europe, he located in Forsyth and entered into partnership with the late Dr. R. L. Roddey. After practicing here for some time, he removed to Dalton, where he continued to practice medicine up to the time of his recent illness, which dates back to November of last year. Dr. Wright married a daughter of Captain W. L. Lampkin, formerly of Forsyth, but now of Dalton.

We extend to the bereaved family our heartfelt sympathy.

METEOROLOGICAL SUMMARY FOR THE MONTH OF JANUARY, 1885,

ATLANTA.

| | |
|---|---|
| Highest Temperature, January 16th, | 64.0 |
| Lowest Temperature, January 18th, | 13.7 |
| Greatest Daily Range of Temperature, January 1st, | 35 5 |
| Least Daily Range of Temperature, January 21st, | 9.0 |
| Mean Daily Range of Temperature, | 16 9 |
| Mean Daily Dew-point, | 28.4 |
| Mean Daily Relative Humidity, | 66.1 |
| Prevailing Direction of Wind, | East. |
| Total Movement of Wind, | 8847 Miles. |
| Highest Velocity of Wind, and direction, | 37 Miles, West. |
| Number of Foggy Days, | 2 |
| Number of Clear Days, | 13 |
| Number of Fair Days, | 8 |
| Number of Cloudy Days, | 10 |
| Number of Days on which Rain or Snow fell, | 17 |
| Depth of Unmelted Snow on Ground at end of Month, | None. |
| Dates of Frosts, | 2, 3, 8, 13, 17, 18, 19, 22, 26, 27, 28, 29. |

